# Antihyperglycemic and Antilipidemic Effects of the Ethanol Extract Mixture of* Ligularia fischeri* and* Momordica charantia* in Type II Diabetes-Mimicking Mice

**DOI:** 10.1155/2018/3468040

**Published:** 2018-10-02

**Authors:** Hyun Jin Baek, Yong Joon Jeong, Jeong Eun Kwon, Jong Sung Ra, Sung Ryul Lee, Se Chan Kang

**Affiliations:** ^1^Department of Oriental Medicine Biotechnology, College of Life Sciences, Kyung Hee University, Yongin 17104, Republic of Korea; ^2^Genencell Co., Ltd, Yongin 16950, Republic of Korea; ^3^BioMedical Institute, Kyung Hee University, Yongin 17104, Republic of Korea; ^4^Department of Convergence Biomedical Science, Cardiovascular and Metabolic Disease Center, College of Medicine, Inje University, Busan 47392, Republic of Korea

## Abstract

The extract of the* Momordica charantia* fruit (MCE) is recognized as an alternative treatment for diabetes. The extract of* Ligularia fischeri *leaves (LFE) is traditionally used as a folk medicine for treating inflammatory diseases in Korea as well. In this study, we investigated the synergistic effect of MCE combined with LFE on antihyperglycemic and antihyperlipidemic potentials. Based on the *α*-glucosidase inhibitory effect and promotion of adipocyte differentiation in the 3T3-L1 cell line, the MLM was prepared with MCE:LFE (8:2 weight:weight). MLM showed the synergistic effects in the promotion of the glucose uptake rate, suppression of dipeptidyl peptidase-4 (DPP-4) mRNA expression, upregulation of an insulin receptor substrate and glucose transporter type-4 expression, and an increase in insulin-associated signaling in C2C12 cells. In addition, the efficacy of peroxisome proliferator-activated receptor-*γ* agonism and glucose uptake rate by MLM supplementation was significantly enhanced* in vitro*. Then, the antihyperglycemic and antihyperlipidemic effects of MCE, LFE, and MLM at the dose of 50, 100, and 200 mg/kg/day (n = 6 per each group) were determined in streptozotocin (STZ)-insulted mice fed an atherogenic diet (ATH) for 4 weeks. In addition, MLM (50, 100, and 200 mg/kg/day, n = 5 per each group) was supplemented in ATH-fed* db/db* mice for 10 weeks. Compared with MCE or LFE alone, MLM supplementation led to a more significant reduction of glucose levels in both STZ/ATH and* db/db*/ATH mice as well as lowered lipid profiles in STZ/ATH mice. In addition, the stimulation of islet of Langerhans regeneration was more pronounced by MLM supplementation in both mice models. In conclusion, antihyperglycemic and antihyperlipidemic effects were strengthened by the combined extracts of* L. fischeri* and* M. charantia* (MLM) in diabetes-mimicking mice.

## 1. Introduction

Diabetes mellitus (DM) is a chronic metabolic disease characterized by high glucose levels due to insulin insufficiency resulting from the loss of pancreatic *β*-cell function (type 1 DM), insulin tolerance (type 2 DM), or both [1]. Diabetes is a major cause of blindness, kidney failure, heart attacks, stroke, and lower limb amputation [1]. Globally, 387 million people currently have diabetes, and it is projected that this condition will be the seventh leading cause of death worldwide by 2030 [2, 3]. Obesity is a physiological condition resulting from the accumulation of excess body fat. Interestingly, glucose metabolism disorders are common in obesity [4], and increased plasma nonesterified fatty acid (NEFA) levels are an important cause of obesity-associated insulin resistance and cardiovascular disease [5]. The first-line treatment for diabetes is glucose-lowering therapy targeting different aspects of glucose metabolism, including insulin sensitivity (e.g., metformin and thiazolidinediones), insulin secretion and bioactivity (e.g., sulfonylureas, glucagon-like peptide 1 [GLP-1] analogues, dipeptidyl peptidase 4 [DPP-4] inhibitors, and insulin analogues), and modulation of blood glucose levels either by increased excretion (e.g., sodium–glucose cotransporter 2 inhibitors, SGLT2i) or by delaying onset following nutrient digestion (e.g., *α*-glucosidase inhibitors and amylin) [6]. The most common therapies, metformin and sulfonylurea, are safe for use in relation to their effect on bone, whereas less frequent therapies such as thiazolidinediones can increase the risk of fractures [6] or other adverse effects [7]. However, pharmacological approaches have not led to improvements in the consequences of insulin resistance. In addition, there is no established remedy for DM, and therapeutic interventions have focused on reducing glucose levels and preventing or delaying complications through diet, physical activity, medication, regular screening, and treatment [1, 8].

The concept of food as medicine is a central theme in dietetic and nutritional sciences [9, 10]. Along with mainstream Western medical treatments, complementary and alternative medicines involving the use of herbs and other dietary supplements have been used as alternatives to treat type 2 DM (T2DM) [9]. Synergy occurs if two or more herbal ingredients mutually enhance each other's effect more significantly than the simple sum of these ingredients [11–13]. Theoretically, optimal dose blending of plant extracts possessing glucose-lowering activity and alleviating obese conditions will be helpful in minimizing adverse effects along with maximizing beneficial outcomes. The extract of* Momordica charantia* fruit (MCE) is widely used as an alternative antidiabetic treatment [3, 14] and also attenuates metabolic changes in experimental obesity [15]. The leaves of* Ligularia fischeri *are used as an edible herb in Korea, and* L. fischeri* extract (LFE) has been used as a traditional medicine for the treatment of inflammatory and infectious diseases as well [16, 17]. However, the antidiabetic potential of LFE has not been well established. In this study, we determined the effects of LFE and MCE on* in vitroα*-glucosidase inhibitory activity and peroxisome proliferator-activated receptor *γ* (PPAR*γ*)-promoting activity [18] based on adipocyte differentiation rates. Then, MLM was prepared with MCE/LFE (8:2 weight/weight) and the synergistic effects of MLM on the hypoglycemic, hypolipidemic, and pancreatic *β*-cell regeneration properties compared to the original MCE and LFE were investigated. First, changes in protein or mRNA levels of molecules involved in insulin-associated signaling pathways such as insulin receptor substrate (IRS), glucose transporter type-4 (GLUT-4), Akt, and PPAR*γ* were assessed in the presence or absence of MCE, LFE, and MLM either in the C2C12 or in the 3T3-L1 cell lines. Subsequently, the antidiabetic effects of MCE, LFE, and/or MLM were determined by supplementation both in mice challenged with streptozotocin (STZ) or in monogenic* db/db* mice fed atherogenic diet (ATH) for 4 and 10 weeks, respectively. Finally, changes in body weight, organ weight, biochemical profile, and histochemical properties of the islets of Langerhans were evaluated.

## 2. Materials and Methods

### 2.1. Chemicals and Reagents

Dulbecco's modified Eagle's medium (DMEM) was obtained from Gibco (Grand Island, NY, USA). Bovine calf serum (BCS), penicillin (100 units/mL)/streptomycin (100 *μ*g/mL), TRIzol, and fetal bovine serum (FBS) were purchased from Invitrogen (Carlsbad, CA, USA). *α*-Glucosidase, acarbose, dexamethasone, and streptozotocin (STZ) were obtained from Sigma (St. Louis, MO, USA). The AdipoRed™ assay reagent was purchased from Lonza (BA, Switzerland). 2-[N-(7-nitrobenz-2-oxa-1,3-diazol-4-yl)amino]-2-deoxy-D-glucose (2-NBDG) was obtained from Thermo Fisher Scientific (Carlsbad, CA, USA). All antibodies were purchased from Cell Signaling Technologies (Beverly, MA, USA).

### 2.2. Preparation of LFE, MCE, and MLM

The leaves of* L. fischeri* and fruit of* M. charantia* were purchased from Jinburyeong (Inje, Korea) and Namyangju-si (Namyang-Ju, Korea) rural markets, respectively. Both were identified by Prof. Kang Se Chan, Kyung Hee University (Yongin, Korea). Two voucher specimens were deposited in the herbarium at Gachon University. The 30% ethanol extract showed the best results when the *α*-glucosidase inhibitory assay and adipocyte differentiation activity were examined by extracting 0 ~ 100% at 10% intervals using ethanol (Supplement Table S1). Therefore, MCE and LFE were prepared by extraction with 30% ethanol for 24 h at 25 ± 5°C. Each extract was then filtered through Whatman Qualitative No. 1 filter paper and concentrated using a rotary evaporator (Eyela, Tokyo, Japan) at 40°C. Both MCE and LFE were freeze-dried using a freeze drier (IlShin Bio Base, Gwangju, Korea). The antidiabetic activity according to the various mixing ratios of MCE and LFE (weight/weight) was confirmed by a preliminary animal test and the best effect was obtained at the mixing ratio of 8:2 (Supplement Table S2). Here, MLM was prepared with MCE and LFE (8:2; weight:weight).

### 2.3. Cell Lines

3T3-L1 mouse fibroblast cell and C2C12 mouse myoblast cells were obtained from American Type Culture Collection (ATCC, Manassas, VA, USA). 3T3-L1 cells were grown in DMEM supplemented with 10% BCS and penicillin/streptomycin in a humidified incubator at 37°C and 5% CO_2_. C2C12 cells were grown in DMEM supplemented with 10% FBS and 1% penicillin/streptomycin.

### 2.4. *α*-Glucosidase Inhibition

The* in vitroα*-glucosidase inhibition assay was performed with an ELISA reader at 405 nm, with acarbose as a specific inhibitor. Briefly, 10 *μ*L of each of MCE, LFE, and MLM at given doses was incubated with a reaction mixture containing 79 *μ*L of sodium phosphate buffer (0.1 M, pH 6.8), 10 *μ*L of para-nitrophenyl glucopyranoside (50 mM), and 1 *μ*L of *α*-glucosidase (0.5 U/mL) at 37°C for 30 min. After the addition of 200 *μ*L of stop buffer (2 M NaOH), the optical density was measured with an ELISA reader (TECAN, Männedorf, Switzerland) at 405 nm.

### 2.5. Adipocyte Differentiation Assay

3T3-L1 preadipocytes were seeded onto 96-well plates (SPL, Seoul, Korea) under the growth conditions described above (1 × 10^4^ cells/well). To induce differentiation, 2-day postconfluent 3T3-L1 cells (designated day 0) were fed DMEM containing 10% FBS, 1 *μ*M dexamethasone (Sigma), and 0.5 mM 3-isobutyl-1-methylxanthine (Sigma) for the following 2 days. Cells were then further cultured with DMEM supplemented with 10% FBS every other day in the presence of MCE, LFE, and MLM (5, 10, and 25 *μ*g/mL). Rosiglitazone (100 *μ*g/mL) was used as a positive control for adipocyte differentiation. At the end of the experiment, AdipoRed™ staining was performed to measure the rate of adipocyte differentiation by measuring fluorescence absorbance. Briefly, the cells were washed twice with phosphate-buffered saline (PBS, pH 7.2), fixed with 4% formaldehyde (Sigma) at room temperature for 4 h, and stained with AdipoRed™ assay reagent (Lonza, BA, Switzerland) for 10 min. The plate was then placed in a fluorimeter (TECAN, Männedorf, Switzerland), and the fluorescence was measured with excitation at 485 nm and emission at 535 nm. Images of the cells were also obtained using a fluorescence microscope (OLYMPUS, Japan) at 488 nm.

### 2.6. 2-NBDG Uptake Assay

Glucose uptake activity was analyzed by measuring the uptake of 2-NBDG, a fluorescent D-glucose analogue. C2C12 cells were seeded on 96-well plates (1 × 10^3^ cells/well) and cultured to 70% confluence in DMEM medium. Then, cells were switched to myoblast differentiation medium containing DMEM supplemented with 2% horse serum, which was replaced every 2 days. Fully differentiated cells were incubated for 24 h in serum-free medium and then treated with 2-NBDG (50 nM) in the presence or absence of MCE, LFE, and MLM at given doses for 24 h. Insulin (100 nM) was used as a positive control for the glucose uptake assay. After washing three times with ice-cold PBS, the intracellular uptake of 2-NBDG was measured using a fluorimeter at excitation and emission wavelengths of 485 and 535 nm, respectively.

### 2.7. Quantitative RT-PCR Analysis

The total RNA from each sample was extracted using TRIzol reagent (Invitrogen) following the manufacturer's instructions. The RNA concentration and quality (260:280 ratio) were assessed using a NanoDrop instrument (Thermo Scientific, Wilmington, DE, USA). Reverse transcription was performed with 0.5 *μ*g of total RNA to generate double-stranded complementary DNA using a PrimeScript™ II 1st Strand cDNA Synthesis Kit (Takara, Japan). Quantitative real-time PCR (qRT-PCR) reactions were performed on a MX3005P (Stratagene, USA). The primers used in the experiments are shown in Table 1. For qRT-PCR, SYBR Premix Ex Taq II (Takara, Japan) was used. The final volume of the reaction was 25 *μ*L, containing 2 *μ*L of cDNA template, 12.5 *μ*L of Master Mix, 1 *μ*L of each primer (10 *μ*M stock solution), and 8.5 *μ*L of sterile distilled water. The thermal cycling profile consisted of a preincubation step at 95°C for 10 min, followed by 40 cycles of 95°C (15 s) and 60°C (60 s). The qRT-PCR data were normalized by the housekeeping gene *β*-actin and expressed as a ratio relative to the untreated control.

### 2.8. Western Blotting

Western blots were performed as previously described [19]. Briefly, cells were washed twice in cold PBS and lysed in a protein extraction solution (iNtRON Biotechnology, Korea) containing a protease inhibitor cocktail (Sigma) for 1 h. The protein concentration was measured by the Bradford method. Sixty micrograms of each sample was electrophoresed on 10% sodium dodecyl sulfate–polyacrylamide gels and transferred to nitrocellulose membranes (BioRad, Hercules, CA, USA). After blocking the membranes with 5% skimmed milk in Tris-buffered saline/Tween-20 (TBST) for 2 h, the target proteins were probed with the appropriate primary antibodies. The blots were washed with TBST and incubated with secondary antibody for 1 h at room temperature. The blots were developed using an enhanced chemiluminescence kit (DoGen, Seoul, Korea). The blots were reprobed with an antibody against *β*-actin, the protein loading control. The signals were detected by a UV trans-illuminator according to the manufacturer's specifications (DAIHAN Scientific Co., Seoul, Korea), and the density of each protein was quantified.

### 2.9. Animals and Study Design

All animals received humane care. The experimental animal facility and study protocols (GIACUC-R2015002) were approved by the Animal Care and Use Committee of Gachon University. All experimental procedures were undertaken in compliance with the Guide for the Care and Use of Laboratory Animals (National Institutes of Health, Bethesda, MD, USA) and the National Animal Welfare Law of the Republic of Korea. C57BLKS/J male mice (lar-Leprdb/Leprdb, 4 weeks old, 13-18 g body weight) were obtained from Central Lab, Animal Inc. (Seoul, Korea) and imprinting control region mice (ICR, 4 weeks old, 15-20 g body weight) were obtained from SLC Inc. (Shizuoka, Japan). The mice were maintained in a controlled environment of 22 ± 1°C and humidity of 50 ± 10% with a 12 h light-dark cycle and were provided with tap water daily. After acclimation, the mice were housed separately in cages and were familiarized with the testing procedures.

### 2.10. Experimental Animals and Diets

**Figure 1:**
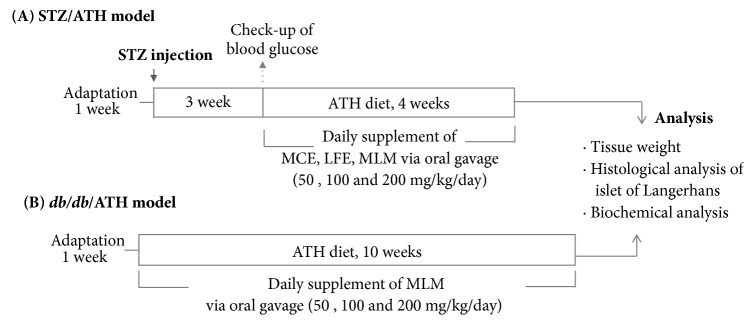
Experimental design of animal study. MCE, ethanol extract of* Momordica charantia* fruit; LFE, ethanol extract of* Ligularia fischeri leaves; *MLM, 8:2 mixture of MCE and LFE; STZ, streptozotocin.

Mice were fed either a chow diet (2018S Teklad Global 18% Protein Rodent diet; Envigo, Madison, WI, USA) or an atherogenic diet (ATH diet, D12336; Research Diets, Inc, New Brunswick, NJ, USA) which is a purified diet matched to the Paigen diet for atherosclerosis research [20]. Two mouse models mimicking T2DM were used in this experiment (Figure 1). For the STZ/ATH diet-induced diabetic mouse model [21], mice were fed an ATH diet after treatment with the pancreatic *β*-cell destructing compound STZ to mimic an obesity-associated diabetic condition. Briefly, ICR mice were allowed to fast for 4 h and then received either the vehicle or 100 mg/kg STZ (in 0.1 M citrate-phosphate buffer, pH 4.5) via intraperitoneal injection [22]. After three weeks, the STZ-injected mice were randomly divided into 10 groups of six animals and were fed with ATH diet with or without treatments for 4 weeks (Figure 1). The other model used was the monogenic* db/db* mouse (C57BL/KsJ) exhibiting characteristics of obesity, hyperinsulinemia, and hyperglycemia. Male* db/db* mice were randomly divided into 4 groups of five animals and were fed with ATH diet with or without MLM treatments for 10 weeks (Figure 1). An appropriate dosing volume of saline or extract was determined after daily weighing. Vehicle, MCE, LFE, and MLM were daily delivered by oral gavage during experimental time. Intragastric delivery of saline or extract was carefully performed by a well-trained researcher to minimize animal stress. During the experiment, weight gain as well as food and water intake calculated by collecting and weighing uneaten food and water was recorded twice per week.

### 2.11. Biochemical Analysis and Determination of Tissue Weight of the Liver, Pancreas, and Epididymal Fat

At the end of the experiment, all animals were fasted for 6 h, and blood was collected from the abdominal vena cava under anesthesia. Blood was centrifuged at 3000 ×* g* for 10 min at 4°C to obtain serum. The serum concentrations of glucose, total cholesterol (T-Chol), triglycerides (TG), NEFA (nonesterified fatty acid, Biocompare, San Francisco, CA, USA), glycated hemoglobin (HbA1c, Abbott IMx Glicohemoglobin; Abbott Laboratories, Amadora, Portugal), and insulin (Biocompare, San Francisco, CA, USA) were enzymatically measured using commercial kits. The liver, pancreas, and epididymal fat were extracted, cleaned with sterile 0.9% NaCl solution, blotted dry with filter paper, and then weighed.

### 2.12. Histological Analysis of the Islets of Langerhans

Animals were sacrificed under anesthesia, and microscopic analysis was carried out on the collected islets of Langerhans. The isolated pancreas was fixed with 10% formalin. The specimens were further fixed with 10% neutral formalin, and histotomy sections of 4 *μ*m thickness were prepared in accordance with a general histological method. The slides were then stained with hematoxylin & eosin dye (H&E), and histopathological inspection was performed with a photomicroscope [23].

### 2.13. High-Performance Liquid Chromatography Analysis

High-performance liquid chromatography (HPLC) analysis was conducted on an LC-20AD (Shimadzu, Japan) fitted with a PDA detector. SkyPack C18 column was 5 *μ*m, 250 mm × 4.6 mm. All solvents used for analysis were HPLC grade and were obtained from J.T. Baker (Phillipsburg, NJ, USA). The mobile phases of mixed solutions (0.1% phosphoric acid:acetonitrile = 2:98) were eluted at a flow rate of 1 mL/min. The UV detector was fixed at 272 nm.

### 2.14. Statistical Analysis

Data are expressed as mean ± SEM (standard error of the mean; in* in vitro* study) or mean ± SD (standard deviation; in animal study). Significance was determined by one-way analysis of variance (ANOVA) followed by a modified* t*-test with Bonferroni correction for comparisons between individual groups using SPSS, version 12 (SPSS Inc., Chicago, IL, USA), where* p* < 0.05 was considered significant.

## 3. Results

### 3.1. Inhibitory Effects of LFE, MCE, and MLM on *α*-Glucosidase Activity

*α*-Glucosidase is an enzyme that catalyzes the final step in the digestion of carbohydrates, converting them to glucose. In diabetic patients, *α*-glucosidase activity is elevated, resulting in increased in blood sugar when carbohydrates are consumed. As shown in Table 2, MCE did not significantly inhibit *α*-glucosidase at concentrations of up to 100 *μ*g/mL, whereas LFE showed a significant inhibitory effect on *α*-glucosidase in a dose-dependent manner. Interestingly, MLM showed stronger inhibitory activity against *α*-glucosidase compared with either MCE or LFE alone. These results suggest that the enhanced *α*-glucosidase inhibitory effect of MLM may be due to an unidentified synergistic effect between MCE and LFE.

### 3.2. Adipocyte Differentiation-Promoting Effects of MCE, LFE, and MLM

**Figure 2:**
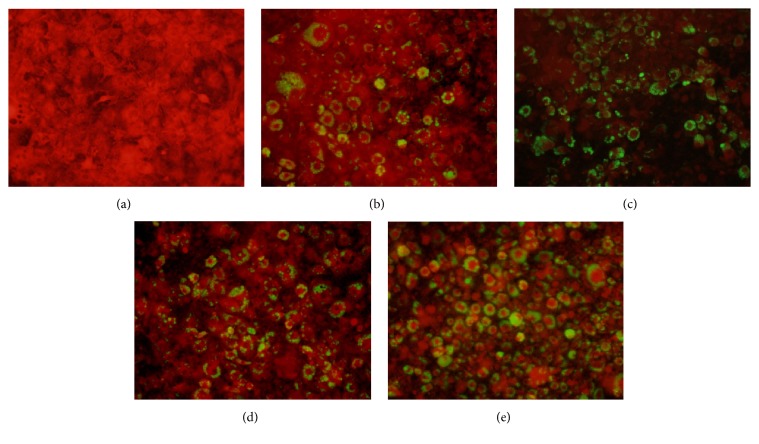
Fluoroscopy of adipocyte differentiation in 3T3-L1 cells. CON (a), MCE (b), LFE (c), MLM (d), and Rosiglitazone (e). MCE, ethanol extract of* Momordica charantia* fruit; LFE, ethanol extract of* Ligularia fischeri leaves; *MLM, 8:2 mixture of MCE and LFE.

We used AdipoRed reagent which can fluorescently stain differentiated adipocytes and the intensity of fluorescence was investigated by fluorescence microscopy (Table 3 and Figure 2). The MCE supplement greatly promoted adipocyte differentiation in 3T3-L1 preadipocytes in a dose-dependent manner. However, LFE was less effective in promoting adipocyte differentiation. Similar to *α*-glucosidase inhibition (Table 2), the promotion of adipocyte differentiation by MLM was also greatly enhanced. Based on these results, we suggest that unknown synergistic effects involving both *α*-glucosidase inhibition and promotion of adipocyte differentiation may have occurred when MCE and LFE were combined; this synergy may provide additional *α*-glucosidase inhibitory activity and promotion of adipocyte differentiation, both of which are beneficial in controlling hyperglycemia and obesity. However, the mechanisms involved in this MLM synergistic effect remain unclear.

### 3.3. Effects of LFE, MCE, and MLM on Glucose Uptake in C2C12 Cells

**Figure 3:**
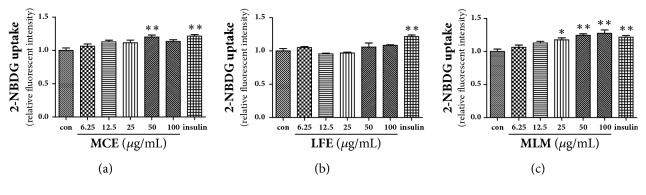
2-NBDG uptake assay in C2C12 cells. MCE (a), LFE (b), and MLM (c). Data are the mean ± SEM. *∗ p* < 0.05, *∗∗ p* < 0.01 versus the untreated control. 2-NBDG, 2-[N-(nitrobenz-2-oxa-1,3-diazol-4-yl) amino]-2-deoxy-d-glucose; MCE, ethanol extract of* Momordica charantia* fruit; LFE, ethanol extract of* Ligularia fischeri leaves; *MLM, 8:2 mixture of MCE and LFE.

Lowering blood glucose levels may be achieved by enhanced uptake of glucose into muscle cells, at least in part. Thus, the effects of MCE, LFE, and MLM on the glucose uptake rate were determined in C2C12 cells after differentiation. As shown in Figure 3, the MCE supplement could stimulate glucose uptake at a concentration of 50 *μ*g/mL, in contrast to LFE; the glucose uptake rate in C2C12 cells was increased by MLM supplementation, even at a concentration of 25 *μ*g/mL. These results suggest that the antihyperglycemic effect of MCE may be augmented in combination with LFE.

### 3.4. Effects of LFE, MCE, and MLM on the mRNA Expression Levels of DPP-4 and GLP-1R

**Figure 4:**
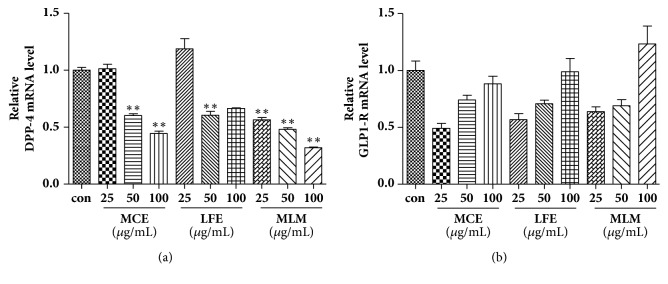
Effects of MCE, LFE, and MLM on the mRNA expression of DPP-4 and GLP-1R in C2C12 cells. DPP-4 (a) and GLP-1R (b). The mRNA expression levels of each gene were normalized with GAPDH. Then control value was set at 1.0 and expressed as a relative ratio of control value. Data are the mean ± SEM. *∗∗ p* < 0.01 versus the untreated control. MCE, ethanol extract of* Momordica charantia* fruit; LFE, ethanol extract of* Ligularia fischeri leaves; *MLM, 8:2 mixture of MCE and LFE.

Suppression of the expression of dipeptidyl peptidase-4 (DPP-4), which rapidly degrades endogenous glucagon-like peptide 1 (GLP-1), and/or upregulation of glucagon-like peptide 1 receptor (GLP-1R), leads to an increase in the secretion and bioactivity of insulin [6]. The mRNA expression level of DPP-4 in C2C12 cells was highly suppressed by both MCE and LFE supplementation at above 50 *μ*g/mL (Figure 4(a)). The mRNA expression level of DPP-4 was highly suppressed by a relatively low concentration of MLM supplementation compared to either MCE or LFE alone. The expression level of GLP-1R mRNA showed an increasing tendency, but there was no statistical significance (Figure 4(b)).

### 3.5. Effects of LFE, MCE, and MLM on the mRNA Expression Levels of GLUT-4, IRS-1, and PPAR*γ* in 3T3-L1 Cells

**Figure 5:**
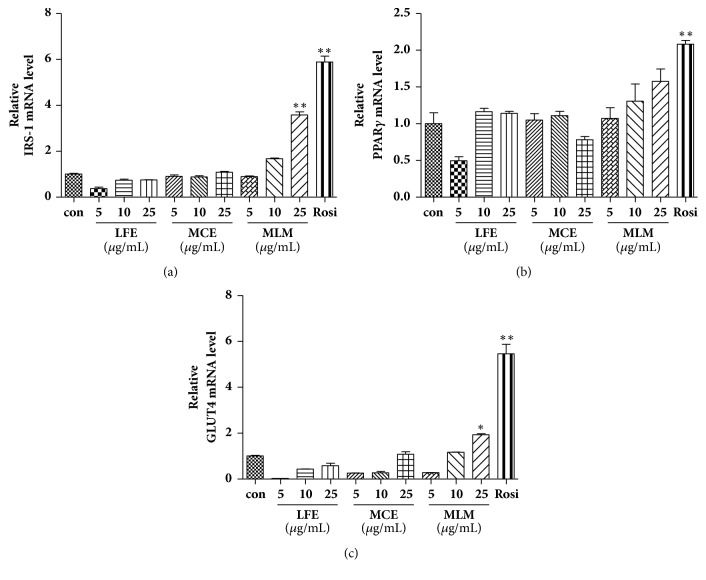
Effects of MCE, LFE, and MLM on the mRNA expression of IRS-1, GLUT-4, and PPAR*γ* in 3T3-L1 cells. IRS-1 (a), GLUT-4 (b), and PPAR*γ* (c). The mRNA expression levels of each gene were normalized with GAPDH. Then control value was set at 1.0 and expressed as a relative ratio of control value. Data are the mean ± SEM. *∗ p* < 0.05, *∗∗ p* < 0.01 versus the untreated control. MCE, ethanol extract of* Momordica charantia* fruit; LFE, ethanol extract of* Ligularia fischeri leaves; *MLM, 8:2 mixture of MCE and LFE.

PPAR*γ* is distributed mainly in adipose tissue and skeletal muscle and plays a critical role in regulating glucose metabolism [18]. IRS-1 is one of the key signaling proteins involved in the insulin signaling pathway. The glucose uptake rate in muscle or adipocytes is controlled by the actions of insulin receptor substrate 1 (IRS-1), PPAR*γ*, and GLUT-4. In the presence of the PPAR*γ* agonist rosiglitazone, the mRNA expression levels of IRS-1, GLUT-4, and PPAR*γ* in 3T3-L1 cells showed marked increases (Figure 5). Neither MCE nor LFE increased the mRNA expression levels of IRS-1 (Figure 5(a)) or GLUT-4 (Figure 5(b)), which are transcriptional targets for PPAR*γ*. However, MCE supplementation increased the mRNA expression levels of IRS-1 and GLUT-4 at a concentration of 25 *μ*g/mL. The mRNA expression level of PPAR*γ* was increased without statistical significance in the presence of MLM. Neither MCE nor LFE alone affected the mRNA expression level of PPAR*γ* (Figure 5(c)).

### 3.6. Effects of LFE, MCE, and MLM on the Activation of Insulin-Associated Signaling in 3T3-L1 Cells

**Figure 6:**
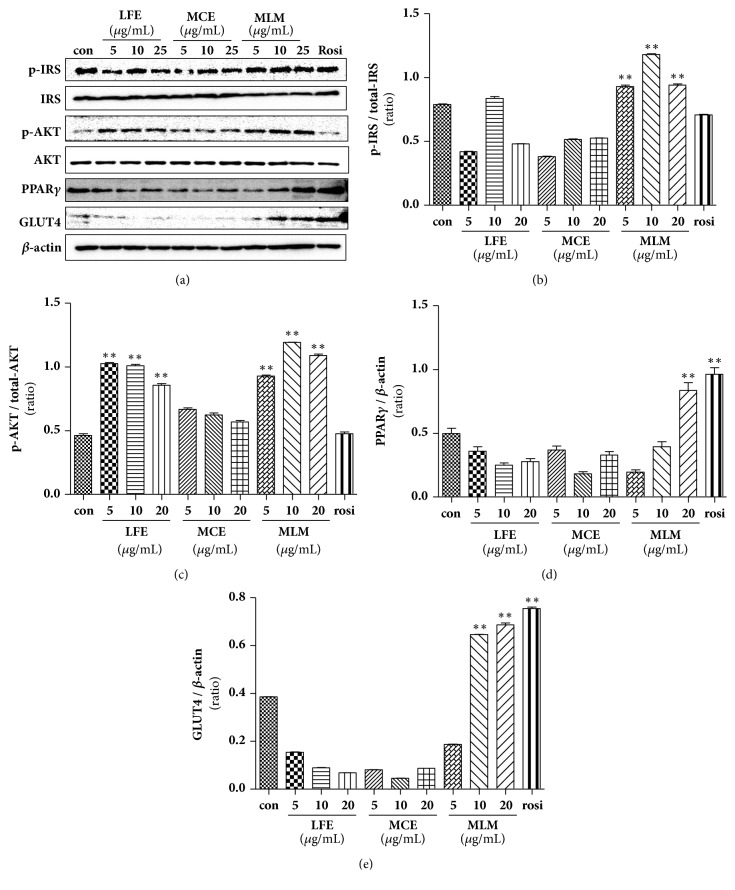
Effects of MCE, LFE, and MLM on the protein expression of IRS, AKT, GLUT-4, and PPAR*γ* in 3T3-L1 cells. (a) A representative image of an immunoblot. (b) Phospho (p)-IRS/total IRS level. (c) p-Akt/total AKT. (d) Protein level of GLUT-4. (e) Protein level of PPAR*γ*. Data are the mean ± SEM. *∗∗p* < 0.01 versus the untreated control. MCE, ethanol extract of* Momordica charantia* fruit; LFE, ethanol extract of* Ligularia fischeri leaves; *MLM, 8:2 mixture of MCE and LFE.

To determine the effects of MCE, LFE, and MLM on the activation of insulin-associated signaling, the protein expression levels of GLUT-4, PPAR*γ*, and the phosphorylated forms of both IRS and Akt were determined by immunoblots using 3T3-L1 cell lysates. As shown in Figure 6, rosiglitazone treatment increased the protein expression levels of PPAR*γ* and GLUT-4, although IRS and Akt were not activated, as determined by the ratios relative to their phosphorylated forms. The protein expression patterns of IRS, GLUT-4, and PPAR*γ* were similar to the RT-PCR results shown in Figure 5. MCE treatment did not affect the protein expression of PPAR*γ*, GLUT-4, or the phosphorylated forms of IRS and Akt. LFE treatment showed a similar pattern to MCE, except for Akt phosphorylation. Interestingly, the protein expression levels of PPAR*γ*, GLUT-4, and the phosphorylated forms of IRS and Akt greatly increased with MLM treatment. These results suggest that MCE or LFE may have a low capacity for improving insulin sensitivity, but the combination of the two may have a synergistic effect on insulin sensitivity through activation of IRS and Akt, as well as upregulation of PPAR*γ* and GLUT-4 expression.

### 3.7. Effects of MCE, LFE, and MLM Supplementation on Water Intake, Food Intake, and Food Efficiency Rate in STZ/ATH and Monogenic* db/db*/ATH Mice

Based on the* in vitro* results, the antidiabetic effects of MCE, LFE, and MLM at doses of 50, 100, and 200 mg/kg/day were investigated in two types of T2DM-mimicking mice, STZ-induced diabetic mice, and monogenic* db/db* mice fed ATH diets (Figure 1). As shown in Table 4, STZ/ATH mice showed larger increases in water and food intake rates, which are phenotypic symptoms observed in diabetic conditions, although body weight decreased but without statistical significance. The water and food intake rates were highly suppressed by MCE supplementation, in contrast to LFE supplementation. MCE supplementation suppressed the water intake rate in both STZ/ATH and* db/db*/ATH mice but did not affect the rate of food intake.

### 3.8. Effects of MLM on Liver, Pancreas, and Epididymal Fat Weights in STZ/ATH and* db/db*/ATH Mice

At the end of the experiment, the tissue weights of the liver, pancreas, and epididymal fat were measured for STZ/ATH and* db/db*/ATH mice (Table 5). Treatment with STZ/ATH caused a significant increase in the tissue weight of the liver, and this increase was greatly suppressed by supplementation with MCE and LFE at doses of over 100 mg/kg/day, while MLM suppressed the increase in liver tissue weight at a dose of 50 mg/kg/day. Supplementation with either MCE or LFE suppressed epididymal fat accumulation, but LFE had a more potent effect than MCE. MLM was also strongly suppressed epididymal fat accumulation at a given dose but was less effective than LFE alone. The tissue size of the pancreas in the* db/db*/ATH mice gradually enlarged. Although there was no effect on tissue weight of the pancreas with MCE, LFE, or MLM supplementation in STZ/ATH mice, MLM supplementation at a given dose markedly suppressed the tissue weight of the pancreas in* db/db*/ATH mice.

### 3.9. Histological Observations of the Effects of MLM on Islets of Langerhans from STZ/ATH and* db/db*/ATH Mice

**Figure 7:**
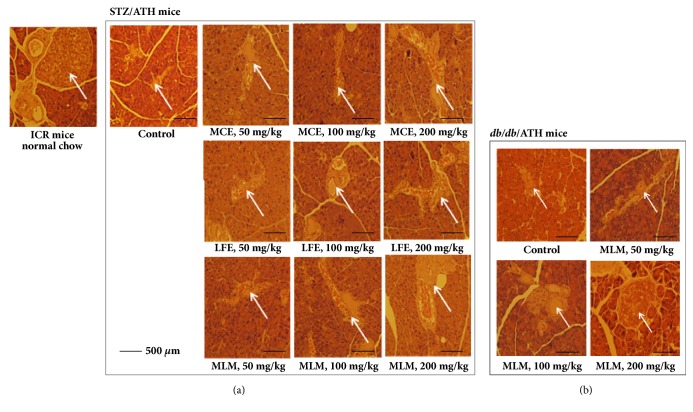
Effects of MCE, LFE, and MLM on the regeneration of islets of Langerhans in either STZ/ATH or* db/db*/ATH mice. ATH, atherogenic diet D12336 (Research Diets, Inc.); MCE, ethanol extract of* Momordica charantia* fruit; LFE, ethanol extract of* Ligularia fischeri leaves; *MLM, 8:2 mixture of MCE and LFE; STZ, streptozotocin (100 mg/kg).

Insulin-secreting *β*-cells in the pancreas account for 65-80% of the islets of Langerhans. Therefore, when treating with STZ which destroys *β*-cells in the pancreas, the size of the islets of Langerhans decreases. Although there was no change in the weight of the pancreas after STZ treatment (Table 5), this does not exclude the destruction of *β*-cells in the islets of Langerhans. When histological changes in the islets of Langerhans were determined in the presence or absence of MCE, LFE, and MLM supplementation, STZ/ATH mice had severe atrophy in the islets of the pancreas (Figure 7(a)). This effect was rescued by treatment with MCE and LFE. The recovery potential in pancreatic islets via MLM supplementation was greater than single treatment with either MCE or LFE. In contrast,* db/db*/ATH mice showed a marked reduction in the size of the islets of the pancreas compared to untreated ICR mice, which could nonetheless be mitigated by supplementation with MLM (Figure 7(b)). These data from STZ/ATH and* db/db*/ATH mice suggest that MCE and LFE may be beneficial to the regeneration of the islets of Langerhans, while the MCE and LFE combination may have a synergistic effect on pancreatic islets.

### 3.10. Effects of MLM on Changes in the Chemical Profiles of STZ/ATH and* db/db*/ATH Mice

At the end of the experiment, biochemical profiles including blood glucose, TG, cholesterol, and NEFA levels were determined in both STZ/ATH and* db/db*/ATH mice. STZ/ATH and* db/db*/ATH mice had high glucose levels of 519.0 ± 48.8 and 563.0 ± 16.0 mg/dL, respectively (Table 6). The STZ/ATH group also had high levels of HbA1c. Since the insulin levels of the STZ/ATH group were unchanged compared with the untreated control (Table 6), the increase in glucose levels was not due to insulin insufficiency. TG, T-Chol, and NEFA levels were significantly higher in STZ/ATH mice than in the untreated control group (Table 6). MCE supplementation strongly attenuated the high glucose and T-Chol levels but was not effective at lowering the levels of TGs and NEFAs. This result may be indirectly connected to the low *α*-glucosidase inhibitory effect (Table 2) and high stimulatory effect on the adipocyte differentiation rate (Table 3). LFE had significant glucose-lowering activity (Table 6). Unlike MCE, LFE significantly suppressed the levels of TG, T-Chol, and NEFA (Table 6). At the same dose, MLM supplementation was more potent in reducing glucose, TG, T-Chol, and NEFA levels in diabetic conditions than either MCE or LFE alone. In* db/db*/ATH mice, MLM supplementation suppressed the elevated glucose levels but was less effective at correcting the lipid profile, likely due to the high levels of insulin in these mice. Collectively, MLM supplementation greatly improved hyperglycemic and hyperlipidemic conditions, which may be associated with combining different sites of action based on the inhibition of *α*-glucosidase activity and promotion of adipocyte differentiation; importantly, a synergistic effect with unidentified mechanisms was apparent.

### 3.11. Chromatogram Analysis

**Figure 8:**
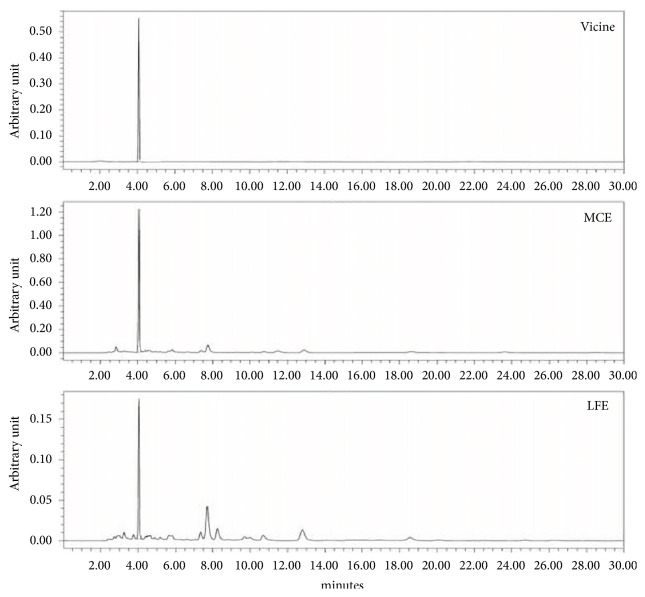
Chromatograms of standard of vicine, MCE, and LFE. HPLC analysis was conducted on a LC-20AD mounted with a PDA detector (272 nm). The common ingredient vicine measured 5.5 mg/g in MCE and 1.3 mg/g in LFE, respectively.

Vicine content was also analyzed as it is common to both LFE and MCE used in this study.  Figure 8 shows the chromatograms of the vicine standard, MCE, and LFE. Linearity was evaluated by building an external calibration curve for vicine at a concentration range of 0.0629 – 1.0 mg/mL and the analytic peak area response was plotted versus its concentration. The results showed that vicine was present at 5.5 and 1.3 mg/g in MCE and LFE, respectively.

## 4. Discussion

In this study, LFE showed a high *α*-glucosidase inhibitory activity (Table 1). MCE did not show significant *α*-glucosidase inhibitory activity but strongly promoted adipocyte differentiation in 3T3-L1 cells. Synergic effects of MLM, which was prepared with MCE/LFE (8:2 weight:weight), were found in** (1)** promotion of the glucose uptake rate in C2C12 cells (Figure 3),** (2)** suppression of DPP-4 mRNA expression (Figure 4),** (3)** upregulation of IRS and GLUT-4 expression (Figure 5),** (4)** increased insulin-associated signaling (Figure 6). In addition to the acceleration of islet of Langerhans regeneration (Figure 7), the enhanced antihyperglycemic and antihyperlipidemic effects were produced by MLM supplementation in STZ/ATH mice and/or* db/db*/ATH mice (Table 6).

In this study, we found that LFE had potent *α*-glucosidase inhibitory activity (Table 2) but was less effective in promoting adipocyte differentiation than MCE (Table 3). Inhibition of *α*-glucosidase activity and increase in the adipocyte differentiation rate were dramatically promoted by MLM treatment* in vitro* compared to either LFE or MCE alone. Enhanced cellular glucose uptake rates that are dominant within muscle and adipose tissues can efficiently lower blood glucose levels in diabetic conditions. In this regard, neither LFE nor MCE alone were effective in promoting glucose uptake in C2C12 cells, but there was a marked improvement in the glucose uptake rate with MLM treatment (Figure 3). In addition, MCE treatment suppressed the mRNA expression level of DPP-4, which negatively regulates blood glucose levels via the degradation of incretins such as a GLP-1. The stimulatory effect of MLM on glucose uptake may be associated with activation of a PPAR*γ* agonist-like effect, resulting in an increase in the mRNA expression levels of IRS and GLUT-4 in 3T3-L1 cells (Figure 5). MLM treatment also led to an increase in the protein expression levels of p-IRS and GLUT-4 (Figure 6); additionally, insulin-associated signaling through IRS and Akt was highly activated by MLM treatment in 3T3-L1 cells (Figure 6). It is unclear how combining LFE with MCE results in strong inhibition of *α*-glucosidase activity and adipocyte differentiation rate; however, this finding implies that there may be unidentified synergistic interactions in MLM. As shown in Figure 8, the presence of vicine in both plant extracts was confirmed, as previously reported. As a result, vicine was not considered to influence the synergistic effect of the two combined extracts. As further work, we intend to identify the *α*-glucosidase inhibitory factors in LFE as well as the major components affecting adipocyte differentiation in MCE.

The fruit of* M. charantia* (bitter gourd or bitter melon) is not only a nutritious vegetable but has also been used in traditional medical practices to treat T2DM [14, 24, 25]. As a nonmedicinal alternative, MCE has been found to be a good functional food for diabetes intervention [14], but its potential as a remedy for diabetes has not been fully determined*. The leaves of L. fischeri *are another edible herb distributed throughout Eastern Asia including Korea, China, and Japan and are used as a traditional medicine for the treatment of inflammatory and infectious diseases, as well as obesity [26, 27]. Here, we demonstrated the antidiabetic potential of LFE based on its ability to decrease blood glucose, TG, T-Chol, and NEFA levels in STZ/ATH mice (Table 6). To increase and/or compensate for the antidiabetic effect of MCE at lower concentrations, MLM was prepared as an 8:2 mixture of MCE and LFE, and its enhanced antidiabetic effects were shown on two different mouse models of T2DM caused by either a high-fat diet with pancreatic insufficiency or insulin resistance-related DM (Table 6). Differently, in diabetes, *β*-cell function within the islets of Langerhans is thought to be severely compromised before disease onset and continues to decrease linearly with time. In STZ/ATH mice, the histological architecture of the islets of Langerhans was severely compromised. Both MCE and LFE supplements attenuated the destruction of the islets of Langerhans in STZ/ATH mice, and the preserving effect of MLM was stronger than each supplement alone (Figure 7). Moreover, the capacity for islet growth in* db/db*/ATH mice was ameliorated by MLM treatment (Figure 7) without altering blood insulin levels (Table 6). This conservative role of MLM in blocking the gradual destruction and aiding in the structural organization of the islets of Langerhans in diabetic conditions may justify the development of a new treatment approach based on MLM. Although there was no difference in body weight among STZ/ATH mice in the presence or absence of treatments, weight gain was observed for the liver and epidermal fat; however, the weight increase in these tissues was largely suppressed in the presence of LFE, MCE, and MLM. In both STZ/ATH and monogenic* db/db*/ATH mice, MLM significantly ameliorated hyperglycemic effects and weight changes in the liver, pancreas, and epidermal fat. Unlike the STZ/ATH model, enlargement of the pancreas associated with the large capacity for islet growth was observed in* db/db*/ATH mice; however, this was suppressed by MLM treatment [28]. Nutrition has been suggested to be important for modulating the expression of genes involved in metabolic pathways related to T2DM pathogenesis [29]. Inversely, correction of the genomic and/or epigenomic changes associated with T2DM pathogenesis may be influenced by food components such as polyphenols, flavonoids, phenolic acids, or other bioactive compounds [29]. In addition to reducing glucose levels, we propose that MLM may be a potentially beneficial food supplement to provide additional regulatory effects on genes involved in diabetic control since it is involved in the downregulation of DPP-4 (Figure 4) and upregulation of IRS, PPAR*γ*, and GLUT-4 expression (Figures 5 and 6). This regulatory effect of MLM on gene expression could be beneficial in long-term control of diabetic conditions compared to simple inhibition of the production and/or ingestion of glucose.

Collectively, both MCE and LFE supplementation lowered blood glucose via different modes of action on diabetic conditions. MLM showed an enhanced inhibitory effect against *α*-glucosidase activity and a stimulatory effect on both adipocyte differentiation and glucose uptake rate. Furthermore, MLM was more efficient at controlling hyperglycemia and the high lipid profile of STZ/ATH and monogenic* db/db*/ATH mice compared to either MCE or LFE alone. MLM supplementation promoted the regeneration of islets of Langerhans and led to positive changes in the expression of genes involved in diabetes. However, the precise underlying mechanisms involved in the synergic effects of combining MCE and LFE remain unclear; thus, future extensive studies will be necessary to elucidate the relevant synergic mechanisms and active ingredients.

## 5. Conclusion

We confirmed that the efficacy of PPAR*γ* agonism and glucose uptake rate by MLM supplementation was significantly enhanced* in vitro*. Consequently, antihyperglycemic and antilipidemic effects of MLM supplementation were greater than with MCE or LFE alone in diabetes-mimicking mice.

## Figures and Tables

**Table 1:** The primer sequences used in quantitative RT-PCR.

Gene name	Primer sequences
*β*-Actin	5′-TGTCCACCTTCCAGCAGATGT-3′ (sense) 5′-AGCTCAGTAACAGTCCGCCTAGA-3′ (antisense)
Peroxisome proliferator-activated receptor *γ* (PPAR*γ*)	5′-CGCTGATGCATGCCTATGA-3′ (sense) 5′-AGAGGTCCACAGAGCTGATTCC-3′ (antisense)
Glucose transporter type-4 (GLUT-4)	5′-AGAGTCTAAAGCGCCT-3′ (sense) 5′-CCGAGACCAACGTGAA-3′ (antisense)
Insulin receptor substrate 1 (IRS-1)	5′-GCCAATCTTCATCCAGTTGC-3′ (sense) 5′-CATCGTGAAGAAGGCATAGG-3′ (antisense)
Glucagon like peptide 1 receptor (GLP-1R)	5′-GCTGCTGGTGGGACACTTGA-3′ (sense) 5′-ATGGTGGCTATCCTGTACTGCTTT-3′ (antisense)
Dipeptidyl peptidase-4 (DPP-4)	5′-CACAGCTATTCCGCACTTGAA-3′ (sense) 5′-TTGTGGATAGCAAGCGAGTTG-3′ (antisense)

**Table 2:** Inhibitory effects of MCE, LFE, and MLM on *α*-glucosidase activity.

Dose (*μ*g/mL)	*α*-glucosidase inhibition rate (%)			
	MCE	LFE	MLM	Acarbose
100	0 ± 0.15	44.40 ± 1.51^*∗∗*^	44.24 ± 1.01^*∗∗*^	48.87 ± 1.05^*∗∗*^
50	0 ± 0.11	41.77 ± 1.80^*∗∗*^	39.02 ± 0.61^*∗∗*^	38.20 ± 1.41^*∗∗*^
25	0 ± 0.14	23.98 ± 0.89	27.91 ± 0.98^*∗∗*^	32.70 ± 1.65^*∗∗*^
12.5	0 ± 0.22	6.84 ± 2.43	23.84 ± 0.79	23.66 ± 0.92
6.25	0 ± 0.15	1.99 ± 1.53	24.97 ± 1.88	31.81 ± 3.31
0	0 ± 0.12			

Values represent the mean ± SEM (n=4). *∗∗p* < 0.01 vs. 0 *μ*g/mL group. MCE, ethanol extract of *Momordica charantia* fruit; LFE, ethanol extract of *Ligularia fischeri leaves; *MLM, 8:2 mixture of MCE and LFE.

**Table 3:** Effects of LFE, MCE, and MLM on the adipocyte differentiation rate in 3T3-L1 cells.

Dose (*μ*g/mL)	Adipocyte differentiation rate (%)			
	MCE	LFE	MLM	Rosiglitazone
100	160.73 ± 1.17^*∗∗*^	101.31 ± 0.27	212.54±3.72^*∗∗*^	323.53 ± 0.71^*∗∗*^
50	142.97 ± 6.73^*∗∗*^	102.82 ± 0.31	178.79 ± 1.60^*∗∗*^	269.24 ± 1.88^*∗∗*^
25	132.75 ± 2.66^*∗∗*^	112.12 ± 0.801	172.51 ± 1.70^*∗∗*^	259.97 ± 7.77^*∗∗*^
12.5	135.60 ± 3.68^*∗*^	124.36 ± 4.32	118.05 ± 1.95	234.06 ± 0.53^*∗∗*^
6.25	106.00 ± 5.33	126.51 ± 1.18	129.94 ± 6.49	245.47 ± 5.75^*∗∗*^
0	100.19 ± 2.21			

Values represent the mean ± SEM. *∗∗p* < 0.01 vs. 0 MCE, ethanol extract of *Momordica charantia* fruit; LFE, ethanol extract of *Ligularia fischeri leaves; *MLM, 8:2 mixture of MCE and LFE.

**Table 4:** Changes in water intake, food intake, and body weight gain in STZ/ATH and *db/db*/ATH mice.

Group	Water intake (mL/day)	Food intake (g/day)	Body weight gain (g)
ICR mice (n = 6 per each group)			
Normal control	4.5 ± 0.37	4.6 ± 0.30	6.4 ± 2.28
STZ/ATH mice (n=6 per each group)			
vehicle	13.9 ± 1.34^*∗∗*^	8.3 ± 0.69^*∗∗*^	3.0 ± 3.14
MCE (mg/kg/day)			
50	11.7 ± 1.12^##^	8.7 ± 0.61	2.4 ± 1.07
100	10.9 ± 1.39^##^	8.3 ± 0.68	2.5 ± 0.36
200	9.9 ± 1.36^##^	7.4 ± 1.06^##^	4.0 ± 0.74
LFE (mg/kg/day)			
50	13.8 ± 1.50	8.2 ± 0.52	1.8 ± 0.84
100	13.6 ± 1.29	9.1 ± 0.95^#^	2.4 ± 0.67
200	13.6 ± 1.53	10.0 ± 1.62^##^	2.4 ± 0.39
MLM (mg/kg/day)			
50	13.0 ± 1.46	6.6 ± 1.02^##^	2.6 ± 1.18
100	11.6 ± 1.37^##^	8.2 ± 0.57	2.8 ± 2.69
200	10.8 ± 1.55^##^	8.4 ± 0.91	3.1 ± 0.87
*db/db*/ATH mice (n=5 per each group)			
vehicle	11.3 ± 1.64	5.7 ± 0.82	25.2 ± 1.91
MLM (mg/kg/day)			
50	10.7 ± 1.52	6.4 ± 0.50^*∗*^	25.0 ± 1.82
100	9.7 ± 1.70^*∗*^	6.3 ± 0.72	22.9 ± 2.77
200	9.2 ± 1.51^*∗∗*^	5.3 ± 0.87	21.1 ± 3.72

Data are the mean ± SD (each group, n=6). *∗p* < 0.05, *∗∗p* < 0.01, vs. the normal control group. ^#^*p* < 0.05, ^##^*p* < 0.01 vs. the STZ/ATH group. ATH, atherogenic diet D12336 (Research Diets, Inc.); MCE, ethanol extract of *Momordica charantia* fruit; LFE, ethanol extract of *Ligularia fischeri leaves; *MLM, 8:2 mixture of MCE and LFE; STZ, streptozotocin (100 mg/kg).

**Table 5:** Changes in relative tissue weight of the liver, pancreas, and epididymal fat in STZ/ATH and *db/db*/ATH mice.

Group	Relative tissue weight (Tissue weight (g) / Body weight (g))		
	Liver	Pancreas	Epididymal fat
ICR mice (each n=6)			
Normal control	0.0343 ± 0.0035	0.0058 ± 0.0003	0.0313 ± 0.005
STZ/ATH mice (n = 6 per each group)			
Vehicle	0.0758 ± 0.0099^*∗∗*^	0.0069 ± 0.0009	0.0224 ± 0.0035^*∗∗*^
MCE (mg/kg/day)			
50	0.0627 ± 0.0026	0.0057 ± 0.0009	0.0119 ± 0.0025^##^
100	0.0617 ± 0.0026^#^	0.0067 ± 0.0003	0.0153 ± 0.0032^#^
200	0.0547 ± 0.0045^##^	0.0065 ± 0.0011	0.0145 ± 0.0019^#^
LFE (mg/kg/day)			
50	0.0632 ± 0.0025	0.0059 ± 0.0011	0.0079 ± 0.0018^##^
100	0.0631 ± 0.0057^#^	0.0067 ± 0.0013	0.0092 ± 0.0022^##^
200	0.0594 ± 0.0072^#^	0.0062 ± 0.0018	0.0083 ± 0.0023^##^
MLM (mg/kg/day)			
50	0.0648 ± 0.0043^#^	0.0065 ± 0.0009	0.0092 ± 0.0028^##^
100	0.0607 ± 0.0108^#^	0.0073 ± 0.0023	0.0129 ± 0.0024^##^
200	0.0594 ± 0.0052^#^	0.0065 ± 0.0008	0.0151 ± 0.0019^#^
*db/db*/ATH mice (n = 5 per each group)			
Vehicle	0.0580 ± 0.0040	0.0060 ± 0.0007	0.0557 ± 0.0041
MLM (mg/kg/day)			
50	0.0602 ± 0.0031	0.0044 ± 0.0005^*∗*^	0.0052 ± 0.0043
100	0.0548 ± 0.0047	0.0041 ± 0.0007^*∗∗*^	0.0485 ± 0.0059
200	0.0513 ± 0.0038^*∗∗*^	0.0035 ± 0.0003^*∗∗*^	0.0470 ± 0.0053^*∗*^

Data are the mean ± SD (each group, n=6). *∗p* < 0.05, *∗∗p* < 0.01 vs. the normal control group. ^#^*p* < 0.05, ^##^*p* < 0.01 vs. the STZ/ATH group. ATH, atherogenic diet D12336 (Research Diets, Inc.); MCE, ethanol extract of *Momordica charantia* fruit; LFE, ethanol extract of *Ligularia fischeri leaves; *MLM, 8:2 mixture of MCE and LFE; STZ, streptozotocin (100 mg/kg).

**Table 6:** Biochemical profiles of STZ/ATH-induced diabetic and *db/db*/ATH mice.

Group	Glucose (mg/dL)	Triglycerides (mg/dL)	Total cholesterol (mg/dL)	HbA1c (%)	NEFA (mEq/L)	Insulin (ng/mL)
ICR mice (each n=6)						
Normal control	56.0 ± 11.7	67.0 ± 9.4	158.0 ± 6.9	4.1 ± 0.19	3.27 ± 0.643	0.26 ± 0.041
STZ/ATH mice ( n = 6 per each group)						
vehicle	519.0 ± 48.8^*∗∗*^	137.0 ± 22.2^*∗∗*^	277.0 ± 24.8^*∗∗*^	8.9 ± 0.96^*∗∗*^	6.62 ± 1.335^*∗∗*^	0.23 ± 0.054
MCE (mg/kg/day)						
50	470.0 ± 34.4	132.0 ± 15.5	202.0 ± 29.1^##^	7.9 ± 0.52	5.63 ± 0.718	0.23 ± 0.028
100	408.0 ± 57.3^##^	127.0 ± 17.6	193.0 ±2 0.0^##^	7.6 ± 1.01	5.57 ± 0.585	0.26 ± 0.048
200	390.0 ± 90.6^#^	131.0 ± 17.9	184.0 ± 27.6^##^	7.5 ± 0.94^#^	4.56 ± 0.685^#^	0.39 ± 0.136^#^
LFE (mg/kg/day)						
50	460.0 ± 66.9	122.0 ± 27.2	193.0 ± 25.0^##^	8.1 ± 0.61	5.22 ± 0.655^#^	0.22 ± 0.046
100	399.0 ± 63.3^##^	103.0 ± 14.0^#^	153.0 ± 12.1^##^	7.5 ± 0.78^#^	4.74 ± 0.731^#^	0.23 ± 0.075
200	388.0 ± 46.0^##^	101.0 ± 17.1^#^	159.0 ± 21.2^##^	7.3 ± 0.50^#^	4.02 ± 0.659^##^	0.25 ± 0.095
MLM (mg/kg/day)						
50	451.0 ± 44.7^#^	132.0 ± 28.7	206.0 ± 26.6^##^	7.9 ± 1.05	5.23 ± 0.604^#^	0.21 ± 0.028
100	382.0 ± 54.9^##^	119.0 ± 32.2	168.0 ± 18.4^##^	7.0 ± 0.75^##^	4.82 ± 0.956^#^	0.22 ± 0.060
200	373.0 ± 64.2^##^	93.0 ± 19.7^##^	149.0 ± 21.5^##^	6.8 ± 0.68^##^	4.14 ± 0.514^##^	0.30 ± 0.067
*db/db*/ATH mice (n = 5 per each group)						
vehicle	563.0 ± 16.0	100.0 ± 8.1	223.0 ± 25.1	-	3.37 ± 0.494	4.04 ± 0.593
MLM (mg/kg/day)						
50	550.0 ± 69.5	115.0 ± 14.7^*∗*^	226.0 ± 36.4	-	3.64 ± 0.582	3.92 ± 0.863
100	503.0 ± 54.7^*∗*^	99.0 ± 7.7	213.0 ± 20.9	-	3.33 ± 0.447	3.28 ± 1.169
200	478.0 ± 36.7^*∗∗*^	90.0 ± 7.2	212.0 ± 25.6	-	3.55 ± 0.547	3.13 ± 1.186

Data are the mean ± SEM (each group, n=6). *∗p* < 0.05, *∗∗p* < 0.01 vs. the normal control group. ^#^*p* < 0.05, ^##^*p* < 0.01 vs. the STZ/ATH group. ATH, atherogenic diet D12336 (Research Diets, Inc.); MCE, ethanol extract of *Momordica charantia* fruit; LFE, ethanol extract of *Ligularia fischeri; *MLM, 8:2 mixture of MCE and LFE; STZ, streptozotocin (100 mg/kg).

## Data Availability

The data used to support the findings of this study are available from the corresponding author upon request.

## Supplementary Materials

Supplementary Materials Supp. 1: effects and mix ratio setting of EtOH extract of used plants,* M. charantia* and* L. fischeri*. Table S1: *α*-glucosidase inhibitory and adipocyte differentiation activities of various EtOH concentration extracts for MCE and LFE. Table S2: blood glucose-lowering efficacy of various mixing ratio of MCE and LFE.
