# Supplementary material for: Antihyperglycemic and Antilipidemic Effects of the Ethanol Extract Mixture of Ligularia fischeri and Momordica charantia in Type II Diabetes-Mimicking Mice
Source: Evid Based Complement Alternat Med. 2018 Oct 2;2018:3468040. doi: 10.1155/2018/3468040 (PMC6189690; doi:10.1155/2018/3468040)
Supplement: Supplementary Materials — Supp. 1: effects and mix ratio setting of EtOH extract of used plants, M. charantia and L. fischeri. Table S1: α-glucosidase inhibitory and adipocyte differentiation activities of various EtOH concentration extracts for MCE and LFE. Table S2: blood glucose-lowering efficacy of various mixing ratio of MCE and LFE. [file 3468040.f1.docx]

## Supplementary Materials

Table S1: α-Glucosidase inhibitory and adipocyte differentiation activity of various concentration of MCE and LFE ethanol extracts.

|  | α-Glucosidase inhibition rate (%) Adipocyte differentiation rate (%) | | | | |
| --- | --- | --- | --- | --- | --- |
|  | MCE | | LFE | MCE | LFE |
| Ethanol extractions (%) | | |  |  |  |
| 0 | NE | | NE | 103.62 ± 0.27 | 106.24 ± 0.21 |
| 10 | NE | | 10.23 ± 0.76 | 104.55 ± 0.19 | 105.25 ± 0.15 |
| 20 | NE | | 22.31 ± 1.32 | 143.62 ± 4.07 | 105.78 ± 0.12 |
| 30 | NE | | 42.31 ± 2.07 | 176.22 ± 4.71 | 102.32 ± 0.32 |
| 40 | NE | | 38.08 ± 1.61 | 151.37 ± 4.86 | 101.99 ± 0.28 |
| 50  60  70  80  90 | NE  NE  NE  NE  NE | | 40.22 ± 1.97  36.23 ± 1.48  29.66 ± 1.20  26.33 ± 1.84  NE | 153.97 ± 5.01  137.24 ± 4.69  148.61 ± 5.08  112.32 ± 3.11  106.34 ± 0.22 | 103.59 ± 0.29  107.64 ± 0.11  102.67 ± 0.26  106.54 ± 0.18  105.38 ± 0.13 |
| 100 | | NE 2.24 ± 0.11 106.18 ± 0.41 103.49 ± 0.19 | | | |
| Acarbose  Rosiglitazone | | 40.68 ± 2.08  298.81±4.01 | | | |

MCE, ethanol extracts of *Momordica charantia* fruit; LFE, ethanol extracts of *Ligularia fischeri* leaves*;* NE, no effect. All samples and positive control treatment concentrations are 100 μg/mL.

Table S2: blood glucose-lowering efficacy of various mixing ratio of MCE and LFE.

In the high-fat diet (HFD)/STZ-induced type 2 diabetic animal model, blood glucose-lowering efficacy was measured after 4 weeks of testing to set the optimum mixing ratio of MCE and LFE. As a result of the test, the best blood glucose-lowering efficacy was measured at a ratio of 8:2 (MCE:LFE) and selected as the final composite material for this research.

|  | Blood glucose (μg/dL) | |  |
| --- | --- | --- | --- |
| MCE:LFE  (weight:weight) | 0 week | 4 week | Inhibition rate (%) |
| Control | 370 ± 31.2 | 504 ± 43.9 | - |
| 0:10 | 376 ± 100.5 | 473 ± 78.3 | 6.1 |
| 1:9 | 357 ± 48.1 | 465 ± 71.7 | 7.7 |
| 2:8 | 352 ± 62.2 | 421 ± 70.5 | 16.4 |
| 5:5 | 369 ± 61.5 | 433 ± 83.6 | 14.0 |
| 8:2 | 341 ± 51.6 | 393 ± 71.0 | 22.1 |
| 9:1 | 367 ± 40.2 | 435 ± 28.5 | 13.6 |
| 10:0 | 363 ± 47.5 | 444 ± 53.5 | 12.0 |

MCE, ethanol extracts of *Momordica charantia* fruit; LFE, ethanol extracts of *Ligularia fischeri* leaves.
